# Antigenic Diversity of Human Norovirus Capsid Proteins Based on the Cross-Reactivities of Their Antisera

**DOI:** 10.3390/pathogens10080986

**Published:** 2021-08-05

**Authors:** Junshan Gao, Yueting Zuo, Liang Xue, Linping Wang, Yanhui Liang, Yueting Jiang, Weicheng Cai, Luobing Meng, Jumei Zhang, Qinghua Ye, Shi Wu, Qihui Gu, Tao Lei, Qingping Wu

**Affiliations:** 1 Guangdong Provincial Key Laboratory of Microbial Safety and Health, State Key Laboratory of Applied Microbiology Southern China, Institute of Microbiology, Guangdong Academy of Sciences, Guangzhou 510070, China; gaojs1011@126.com (J.G.); zytscuter@163.com (Y.Z.); wanglinping625822@163.com (L.W.); liangyh77@126.com (Y.L.); lenmonchai@126.com (W.C.); m13684928006@126.com (L.M.); zhangjm926@126.com (J.Z.); yeqinghua2002@163.com (Q.Y.); wushiloveyou@126.com (S.W.); guqh888@163.com (Q.G.); raytao1983@163.com (T.L.); 2 Department of Laboratory Medicine, First Affiliated Hospital of Guangzhou Medical University, Guangzhou 510120, China; jyting8899@126.com

**Keywords:** antigenic diversity, capsid protein, cross-reactivity, human norovirus

## Abstract

Human norovirus (HuNoV), which is the major causative agent of acute gastroenteritis, has broad antigenic diversity; thus, the development of a broad-spectrum vaccine is challenging. To establish the relationship between viral genetic diversity and antigenic diversity, capsid P proteins and antisera of seven GI and 16 GII HuNoV genotypes were analyzed. Enzyme-linked immunosorbent assays showed that HuNoV antisera strongly reacted with the homologous capsid P proteins (with titers > 5 × 10^4^). However, 17 (73.9%) antisera had weak or no cross-reactivity with heterologous genotypes. Interestingly, the GII.5 antiserum cross-reacted with seven (30.4%) capsid P proteins (including pandemic genotypes GII.4 and GII.17), indicating its potential use for HuNoV vaccine development. Moreover, GI.2 and GI.6 antigens reacted widely with heterologous antisera (n ≥ 5). Sequence alignment and phylogenetic analyses of the P proteins revealed conserved regions, which may be responsible for the immune crossover reactivity observed. These findings may be helpful in identifying broad-spectrum epitopes with clinical value for the development of a future vaccine.

## 1. Introduction

Human norovirus (HuNoV) is the main cause of acute nonbacterial gastroenteritis [1,2]. Its infectious dose is as low as 10–100 virus particles [3], and the virus is easily transmitted through food, water, as well as the vomit and feces of infected persons [4]. Although HuNoV infection is usually self-limiting, often causing nausea, vomiting, diarrhea, and headache, it can be lethal in children, the elderly, and people with a compromised immune system [5,6]. HuNoVs are responsible for approximately 699 million infections and over 200,000 deaths annually [7]. However, to date, vaccines preventing HuNoV infections are still not available.

HuNoV, members of the *Caliciviridae* family, are positive-strand RNA viruses with a 7.5–7.7 kb genome [8], consisting of three open reading frames (ORFs). ORF2 encodes the main structural protein, VP1 [9,10], which includes the N-terminal shell (S) domain and the C-terminal protruding (P) domain [11,12]. The P domain is located outside of the virus particle and contains the receptor-binding region and the main antigenic sites. Using an *Escherichia coli* expression system, the capsid P domain can be expressed to form a particle [13,14] with structure, antigenicity, immunogenicity, and receptor-binding function very similar to those of natural virus particles [15]; thus, this strategy can be used as a substitute for HuNoVs, as it meets the requirements of polyclonal and monoclonal antibody preparation.

Based on their VP1, noroviruses are divided into 10 genogroups (GI–GX) and 48 genotypes [16]. HuNoVs are mainly distributed in genogroups GI, GII, GIV, GVIII, and GIX [17,18], and GII.4 is the predominant global variant [16,19]. Since 2014, GII.17 emerged and became the dominant variant [20]. Zhou et al. [21] found that, among the NoV infections from 2006 to 2016, the infection rates of GII genotype and GI genotype were 95.8% and 11.2%, respectively. However, the infection rate of GI in some areas is higher than that of GII. In recent years, GII.6, GII.17, and GII.2 HuNoVs have successively led to epidemics and outbreaks [22]. Stool samples of patients with gastroenteritis and sewage were monitored in Beijing and Taiwan [23,24]. The results showed that non-GII.4 genotypes, such as GI.2, GI.3, GI.6, GII.1, and GII.3, are dominant strains. Owing to its broad antigenic diversity, the evolutionary direction of HuNoV is difficult to monitor. Therefore, research on the correlation between immunogenicity and genotypes could be helpful for understanding the impact of key amino acids of capsid proteins on the antigenicity of HuNoVs.

Existing HuNoV vaccines showed cross-protection effects [25,26,27,28], but until now they were in phase II clinical trials or under development in pre-clinical research, and there is no HuNoV vaccine in the market. Furthermore, norovirus evolved rapidly; the protection of new emerging strains is unknown. Hence, it is necessary to find the key amino acids of HuNoVs capsid protein. This study aimed to establish the relationship between immunogenicity and genetic diversity of HuNoVs, to better understand the cross-protection between genotypes.

## 2. Results

### 2.1. Phylogenetic Analysis of 23 HuNoV Strains

**Figure 1:**
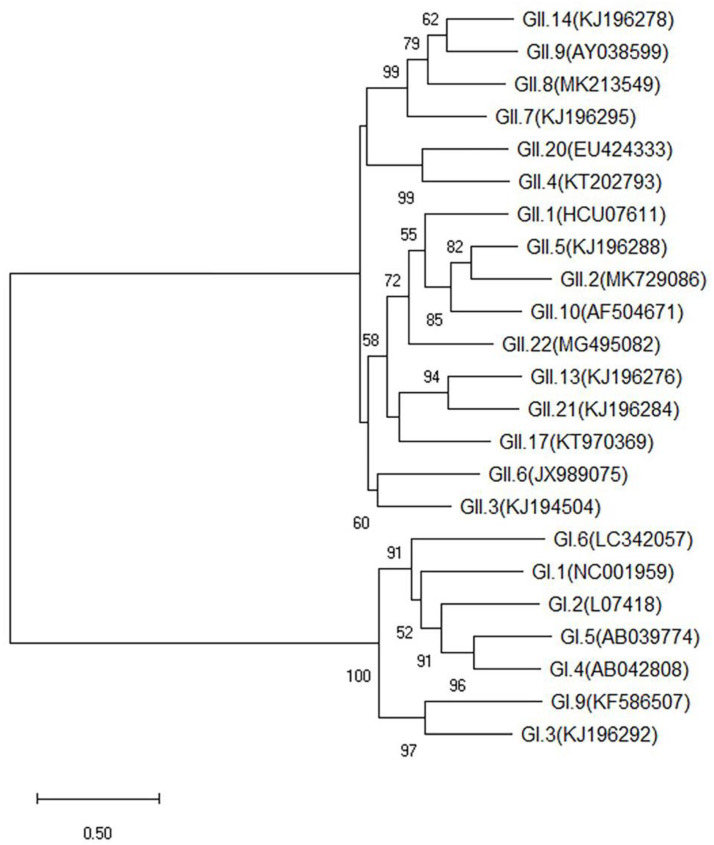
Phylogenetic analysis of the nucleotide sequences of the P domain of 23 human norovirus (HuNoV) genotypes by Maximum Likelihood, with 1000 bootstrap replicates. Bootstrap values are shown next to the branches. The scale bar represents the genetic distance of the nucleotide substitutions per site.

The phylogenetic tree based on the P capsid protein of 7 GI and 16 GII HuNoV genotypes is shown in Figure 1. Among them, the GI.6, GII.2, GII.3, GII.4, GII.8, and GII.17 strains have been previously detected [29,30,31].

### 2.2. Homologous Antigenic Analysis

**Figure 2:**
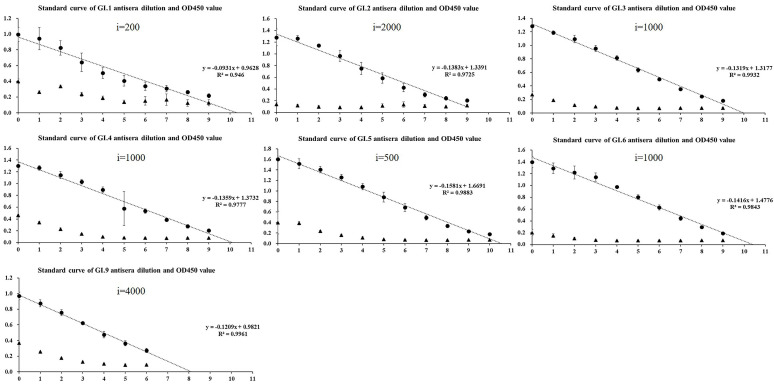
Standard curve of GI norovirus antisera dilution and OD_450_ value determined by enzyme-linked immunosorbent assay.

**Figure 3:**
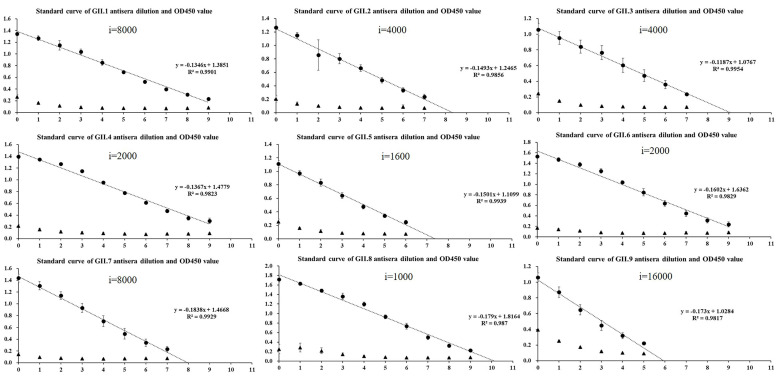

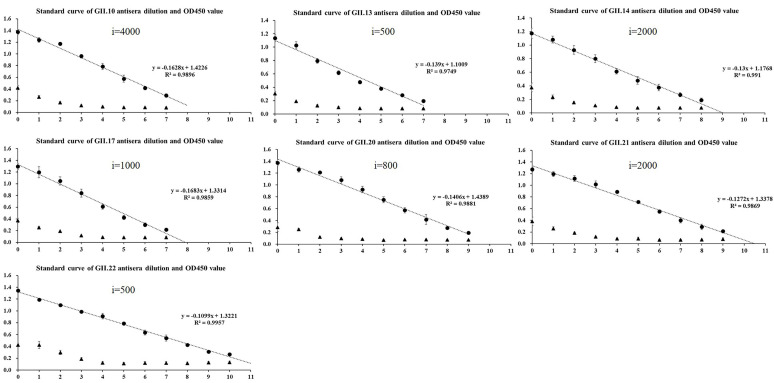
Standard curve of GII norovirus antisera dilution and OD_450_ value determined by enzyme-linked immunosorbent assay.

The enzyme-linked immunosorbent assay (ELISA) revealed that the sera after the third immunization was diluted to 1:10,000, the value of OD_450_ was significantly higher than that of the pre-immune sera (Supplementary Table S1). All antisera after the fourth immunization had strong cross-reactivity with homologous genotypes, and the titration of sera obtained after four immunizations reached up to 51,200–4,096,000. Moreover, except for GI.1 with R^2^ = 0.946, all fit curves had R^2^ > 0.97 (Figure 2 and Figure 3), indicating a good linear relationship between OD_450_ of 0.2–1.4. Moreover, the results of GST partners in this range were all negative.

### 2.3. Heterologous Antigenic Analysis

**Figure 4:**
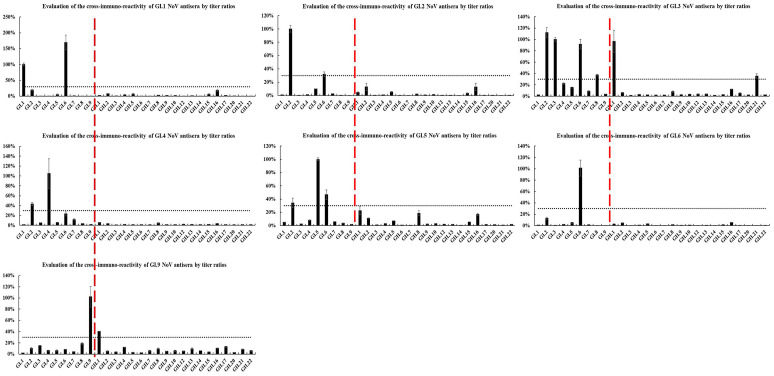
Cross-reactivities of GI HuNoV antisera by titer ratios.

**Figure 5:**
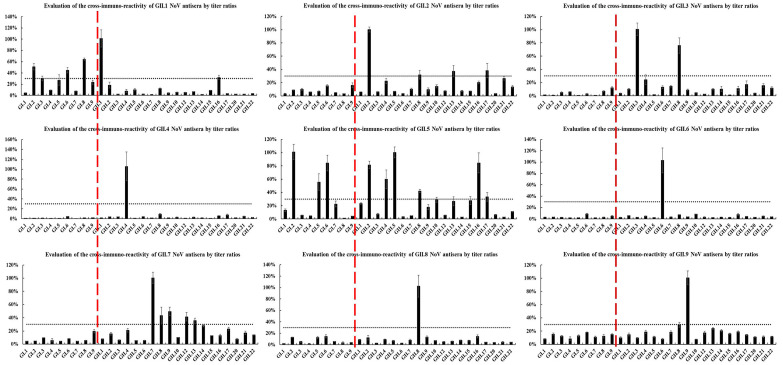

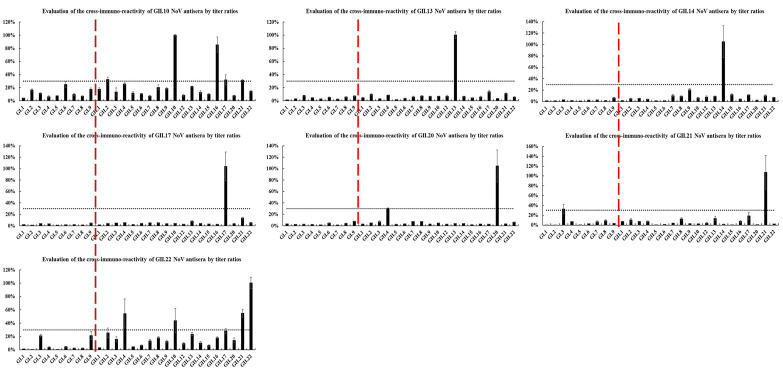
Cross-reactivities of GII HuNoV antisera by titer ratios.

The cross-reactivity of antisera against heterologous HuNoV-genotype P particles was determined by ELISA (Figure 4 and Figure 5). Overall, 17 (73.9%) antisera had weak or no cross-reactivity with heterologous genotypes, among which eight (GI.6, GII.4, GII.6, GII.8, GII.9, GII.13, GII.14, and GII.17) did not react with any other heterologous genotypes. Moreover, GI.3, GII.1, GII.2, GII.10, and GII.22 antisera had moderate cross-reactivity (3 ≤ n < 5), whereas GII.5 antiserum cross-reacted with seven heterologous genotypes. Furthermore, 91.3% of the antisera did not cross-react with heterologous genotypes of different genogroups. In particular, GII.1 did not cross-react with genotypes of the GII genogroup but had moderate cross-reactivity within the GII genogroup (GI.2, GI.3, and GI.6).

GI.2 and GI.6 P particles reacted widely (n ≥ 5), and GII.4, GII.8, GII.17, and GII.21 reacted moderately with antisera of different genotypes (3 ≤ n < 5), whereas the remaining 17 did not react easily (n < 3) with antisera of other genotypes. Of these 17 P particles, 10 did not react with any heterologous antisera. Moreover, 17 (73.9%) genotypes had no reaction, whereas the rest did not easily react (n < 3) with antisera of other genogroups. Interestingly, the P particles of GII.1, GI.3, and GI.5 reacted with GI (GI.3 and GI.9), GII (GII.1 and GII.21), and GII.5 genogroup antisera, respectively, but did not react with other antisera in their respective genogroups.

### 2.4. Antigenic Epitope Analysis

**Figure 6:**
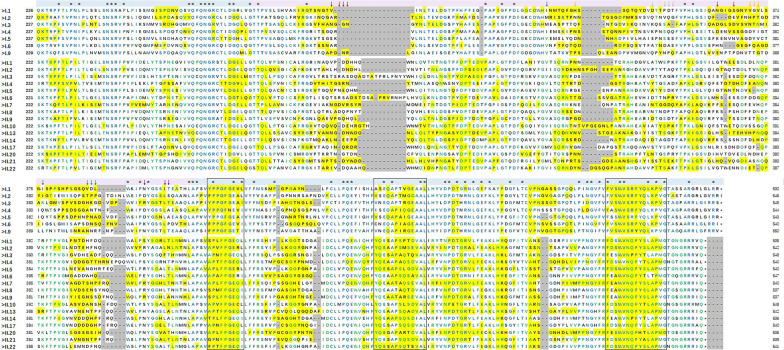
Alignment of P domain amino acid (aa) sequences within antigenic epitopes of 23 human norovirus (HuNoV) strains. Yellow, antigenic site; *, fully conserved aas; black boxes, conserved antigenic regions. Red, blue, orange, green, purple, and grey arrows indicated epitope A, B, C, D, E, and F, respectively. Blue letters represented the conserved amino acids of GI genogroup or GI and GII genogroups, green letters represented conserved amino acids only in the GII genogroup, black letters represented non-conserved amino acids.

The 23 capsid P protein sequences were aligned, then their antigenic site distribution was predicted (Figure 6). The mean pairwise similarity of the 23 sequences was 47.8% (GI: 57.4%; GII: 59.0%), and only 58 sites (16.5%) were conserved across all genotypes, contrasting with 150 (42.6%) and 107 (30.8%) sites that were conserved within the GI and GII genogroups, respectively. Moreover, antigenic site prediction revealed significant differences between genotypes, ranging from 52 to 162 amino acids. Especially in the reported epitopes A/B/C/D/F, no conserved amino acid site was identified. In turn, the sites L412/T413 in epitope E were partially conserved among different genogroups. In addition, the distribution of five antigenic regions in the P1–2 region was relatively consistent (amino acid positions 423–431, 440–448, 500–508, 462–474, and 517–531), and three of them (423–431, 462–474, and 517–531) were conserved across genotypes and even genogroups.

## 3. Discussion

HuNoV infection has become an important public health problem. Currently, effective antiviral drugs and vaccines against HuNoV infection are lacking. Moreover, research is hampered by the difficulty of HuNoV in vitro cultivation and the lack of suitable animal models. Although HuNoV infections have been established in intestinal epithelial cells, immune cells, zebrafish larvae, and a HuNoV culture system, it is still difficult to obtain sufficient and stable viral resources [32,33,34]. Hence, suitable substitutes for HuNoV virus-like and P particles are important. In addition, indicators of HuNoV protective immunity, persistence of natural infection immunity, and cross-protection should be further explored to support the selection of antigen genotypes and evaluation of immune effects in vaccine development.

Herein, the 23 HuNoV P domains evaluated shared exceptionally low sequence similarity, especially within genogroups. Multiple sequence alignment revealed 58 conserved sites (16.5%), and the highly variable antigenic epitopes A/B/C/D. Moreover, antigenic site prediction showed obvious differences between different genotypes, possibly due to the accumulation of mutations during the evolutionary process, which allowed HuNoV strains to thereby evade host immune protection [35,36]. Notably, there were three conserved antigenic sites (amino acid positions 423–431, 462–474, and 517–531) in the P1–2 region, which may have caused the observed immune cross-reactivity. Taken together, these results indicate that a multivalent vaccine might be more effective than a monovalent vaccine in preventing HuNoV infections. These findings warrant further confirmation through mutation, neutralization, blocking experiments, and combined immunization.

Most studies on cross-protection between GI and GII genotypes reported low heterotypic responses between genogroups [37,38]. However, regarding cross-reactivity, contrasting results have been reported [28,39,40]. In this study, the cross-reactivity of antibodies was assessed by detecting the homologous and heterologous genotype antibodies induced by mice immunized with seven GI genotypes and 16 GII genotype P particles. GII.5 immune serum was found to cross-react with seven genotypes of P particles, including the pandemic genotypes GII.4 and GII.17. Nonetheless, no correlation between the amount of P particles reacting with the antisera and the amount of the antisera cross-reacting with the P particles was observed. In fact, a number of them showed the opposite trend. Specifically, GI.6 antiserum had no cross-reactivity with heterologous genotypes, but its P particles reacted widely with antiserum of different genotypes (n = 6). Similarly, GII.5 antiserum had a broad-spectrum cross-reactivity with heterologous genotypes, but its P particles did not react with any other antiserum. Noteworthy, GII.6 and GII.14 antisera both had no cross-reactivity with heterologous genotypes, and their P particles did not react with any other antiserum.

In summary, the relationship between viral genetic diversity and antigenic diversity of HuNoVs was established. These findings may contribute to a better understanding of the cross-protection of antibodies between GI and GII genogroups, and between different genotypes within the same genogroup. Furthermore, these novel insights into HuNoVs features may pave the way for rationally designing a multi-epitope vaccine with a wide range of cross-protection effects to play a role in HuNoV prevention and control.

## 4. Materials and Methods

### 4.1. HuNoV Strains

HuNoV strains used in this experiment were stored in our laboratory listed as follows: GI.1|NC_001959.2, GI.2|L07418.1, GI.3|KJ196292.1, GI.4|AB042808.1, GI.5|AB039774.1, GI.6|LC342057.1, GI.7|KU311161.1, GI.8|KJ196298.1, GI.9|KF586507.1, GII.1|HCU07611, GII.2|MK729086.1, GII.3|KY348697.1, GII.4|KT202793.1, GII.5|KJ196288.1, GII.6|JX989075.1, GII.7|KJ196295.1, GII.8|MK213549.1, GII.9|AY038599.2, GII.10|AF504671.2, GII.12|AB045603, GII.13|KJ196276.1, GII.14|KJ196278.1, GII.15|KJ196290, GII.16|AY772730.1, GII.17|KT970369.1, GII.20|EU424333.1, GII.21|KJ196284.1, and GII.22|MG495082.1.

### 4.2. Expression of P particles

Primer sequences were designed based on the 23 P domains and were synthesized by the Generay company (Shanghai, China), then the CDCRGDCFC amino acid sequence was added to the 5′-end of the reverse primers to promote the formation of P particles [26]. The P domains were amplified by PCR and cloned into a pGEX-4T-1 expression vector, then the recombinant vector was transfected into *E. coli* strain BL21, and P particle expression was induced by incubating the bacterial cells with 0.5 mM isopropyl-β-d-thiogalactopyranoside at 20 °C for 12 h. The recombinant glutathione S-transferase (GST)-tagged proteins were purified using GSTrap FF (Cytiva, Marlborough, MA, USA), according to the manufacturer’s instructions for gel filtration chromatography with the Akta fast performance liquid chromatography system (Cytiva).

### 4.3. Immunization and ELISA

Pathogen-free 8-week-old female BALB/c mice were purchased from the Laboratory Animal Center of Southern Medical University (Guangzhou, China). Each mouse was subcutaneously injected with 30 µg of GST-P protein in Freund’s complete adjuvant (Sigma Aldrich, St. Louis, MO, USA) at week 0 and in Freund’s incomplete adjuvant at weeks 2, 4, and 5. Equivalent phosphate-buffered saline (PBS) was used as negative control. Sera were collected from each mouse at weeks 2, 4, and 5. The animals were bled 1 week after the final immunization.

Sera after the third immunization and the fourth immunization were measured using an ELISA. Ninety-six-well microtiter plates were coated with 0.2 μg/well of GST-P and GST control proteins at 4 °C for 14 h, blocked for 2 h at 37 °C with 200 µL/well of 5% skimmed milk in PBS containing Tween 20 (PBST), then washed three times with PBST. Immunized serum was added to each well at the indicated dilutions, then incubated at 37 °C for 1 h. Samples were assayed in triplicate. The wells were washed four times with PBST, incubated with horseradish peroxidase-conjugated goat anti-mouse IgG antibody (1:3000; Beijing Biosynthesis Biotechnology Co., Beijing, China) for 30 min at 37 °C, and then washed four times with PBST. Each well was incubated with 3,3′,5,5′-tetramethylbenzidine (TIANGEN Biotech Co., Beijing, China) for 10 min, and the reaction was stopped by the addition of 50 µL/well of 1 M H_2_SO_4_. Optical density (OD) values were measured at 450 nm, and those with OD_450_ < 0.2 were considered negative. Antisera titer standard curves were established with OD_450_ values as the ordinate and relative dilution log_2_ x/n as the abscissa (n was the initial antiserum dilution).

### 4.4. Immune Cross-Reactivity

The immune cross-reactivity between antisera and antigens was measured using ELISA. The wells were coated with 23 GST-P proteins as test antigens. Based on the standard curve, the serum dilutions used for cross-reactivity evaluation were determined as OD_450_ within the range of 0.8–1.2 in the homologous antigen analysis. The strength of immune reactivity was calculated as relative titer = (relative dilution/absolute dilution) × 100%, with relative titer > 30% being considered positive. The relative dilution of each antiserum reacting with the heterologous genotypes, and the absolute dilution of the serum reacting with the homologous genotype, were calculated according to the standard curve equation: $y=alog_{2}x/i+b$ (x: actual dilution; i: initial dilution). Moreover, the breadth (how many other genotypes the antisera can bind to) of the antisera cross-reaction was determined as follows: weak, if the number (n) of positive reactions between the antisera and the heterologous genotypes was less than 3 (n < 3); moderate, 3 ≤ n < 5; and strong, n ≥ 5.

### 4.5. Phylogenetic Analysis

Multiple sequence alignment of all P capsid proteins assessed in this study was performed using ClustalW (https://www.genome.jp/tools-bin/clustalw accessed on 8 May 2021), with the default parameters, and phylogenetic trees were constructed using the maximum likelihood method based on the general time reversible model, with 1000 bootstrap replications. All evolutionary analyses were performed using MEGA X (https://www.megasoftware.net accessed on 8 May 2021).

### 4.6. Homology Modeling

Homology models of the P capsid protein structures were established using the Swiss-Model to generate a Protein Data Bank (PDB) file from the P domain amino acid sequence. Spatial epitopes of the 23 capsid proteins were predicted using SEPPA (version 3.0, http://www.badd-cao.net/seppa3/index.html accessed on 12 May 2021). The antigenic epitopes were located according to the reference GII.4-L10 (GenBank: KT202793).

## Data Availability

All data presented in this study are available in this manuscript.

## Supplementary Materials

The following are available online at https://www.mdpi.com/article/10.3390/pathogens10080986/s1, Table S1: Sera after the third immunization determined by enzyme-linked immunosorbent assay.
