# Supplementary material for: Antigenic Diversity of Human Norovirus Capsid Proteins Based on the Cross-Reactivities of Their Antisera
Source: Pathogens. 2021 Aug 5;10(8):986. doi: 10.3390/pathogens10080986 (PMC8398591; doi:10.3390/pathogens10080986)
Supplement: Supplementary file 1 [file pathogens-10-00986-s001.zip › pathogens-1286118-supplementary.pdf]

Table S1 Sera after the third immunization determined by enzyme-linked immunosorbent assay.

Genotypes	Dilution	Blank control (OD450)	Pre-immune sera control (OD450)	Sera after the third immunization (OD450)
I.1	1:10000	0.130	0.079	0.468
I.2	1:10000	0.147	0.082	0.989
I.3	1:10000	0.094	0.069	0.721
I.4	1:10000	0.106	0.075	1.228
I.5	1:10000	0.096	0.080	0.660
I.6	1:10000	0.087	0.088	1.286
I.9	1:10000	0.125	0.079	1.006
II.1	1:10000	0.107	0.090	1.431
II.2	1:10000	0.149	0.099	1.222
II.3	1:10000	0.085	0.080	0.513
II.4	1:10000	0.067	0.097	1.245
II.5	1:10000	0.072	0.055	0.551
II.6	1:10000	0.225	0.101	1.009
II.7	1:10000	0.183	0.083	0.925
II.8	1:10000	0.110	0.069	0.699
II.9	1:10000	0.178	0.098	1.168
II.10	1:10000	0.101	0.078	1.047
II.13	1:10000	0.106	0.085	1.196
II.14	1:10000	0.091	0.071	0.918
II.17	1:10000	0.130	0.076	0.931
II.20	1:10000	0.195	0.116	0.309
II.21	1:10000	0.112	0.079	0.985
II.22	1:10000	0.112	0.079	0.881
